# Nitric oxide exerts protective effects against bleomycin-induced pulmonary fibrosis in mice

**DOI:** 10.1186/s12931-014-0092-3

**Published:** 2014-08-05

**Authors:** Shingo Noguchi, Kazuhiro Yatera, Ke-Yong Wang, Keishi Oda, Kentarou Akata, Kei Yamasaki, Toshinori Kawanami, Hiroshi Ishimoto, Yumiko Toyohira, Hiroaki Shimokawa, Nobuyuki Yanagihara, Masato Tsutsui, Hiroshi Mukae

**Affiliations:** 1 Department of Respiratory Medicine, University of Occupational and Environmental Health, Japan, 1-1 Iseigaoka, Yahatanishiku, Kitakyusyu, 807-8555, Fukuoka, Japan; 2 Shared-Use Research Center, University of Occupational and Environmental Health, Japan, Kitakyusyu, Fukuoka, Japan; 3 Department of Pharmacology, School of Medicine, University of Occupational and Environmental Health, Japan, Kitakyusyu, Fukuoka, Japan; 4 Department of Cardiovascular Medicine, Tohoku University Graduate School of Medicine, Sendai, Japan; 5 Department of Pharmacology, Graduate School of Medicine, University of the Ryukyus, Ryukyus, Okinawa, Japan

## Abstract

**Background:**

Increased expression of nitric oxide synthase (NOS) and an increase in plasma nitrite plus nitrate (NOx) have been reported in patients with pulmonary fibrosis, suggesting that nitric oxide (NO) plays an important role in its development. However, the roles of the entire NO and NOS system in the pathogenesis of pulmonary fibrosis still remain to be fully elucidated. The aim of the present study is to clarify the roles of NO and the NOS system in pulmonary fibrosis by using the mice lacking all three NOS isoforms.

**Methods:**

Wild-type, single NOS knockout and triple NOS knockout (n/i/eNOS^−/−^) mice were administered bleomycin (BLM) intraperitoneally at a dose of 8.0 mg/kg/day for 10 consecutive days. Two weeks after the end of the procedure, the fibrotic and inflammatory changes of the lung were evaluated. In addition, we evaluated the effects of long-term treatment with isosorbide dinitrate, a NO donor, on the n/i/eNOS^−/−^ mice with BLM-induced pulmonary fibrosis.

**Results:**

The histopathological findings, collagen content and the total cell number in bronchoalveolar lavage fluid were the most severe/highest in the n/i/eNOS^−/−^ mice. Long-term treatment with the supplemental NO donor in n/i/eNOS^−/−^ mice significantly prevented the progression of the histopathological findings and the increase of the collagen content in the lungs.

**Conclusions:**

These results provide the first direct evidence that a lack of all three NOS isoforms led to a deterioration of pulmonary fibrosis in a BLM-treated murine model. We speculate that the entire endogenous NO and NOS system plays an important protective role in the pathogenesis of pulmonary fibrosis.

## Introduction

Pulmonary fibrosis is an interstitial lung disease characterized by chronic inflammation and progressive fibrosis of the pulmonary interstitium (alveolar walls and septa, perivascular, perilymphatic and peribronchiolar connective tissues) [1]. It is believed that lung inflammation initiates lung fibrosis, however, the etiological mechanism of this disease has not yet been fully elucidated [2].

Nitric oxide (NO) is gaseous free radical, and is formed from its precursor, L-arginine, by a family of NO synthases (NOSs) with stoichiometric production of L-citrulline [3]. NO plays an important role in maintaining respiratory homeostasis [4],[5]. There are three distinct isoforms of NOS, two of which are constitutive NOSs known as neuronal NOS (nNOS) and endothelial NOS (eNOS), and other is inducible NOS (iNOS). The expression of constitutive NOSs (nNOS and eNOS) has been observed in various types of pulmonary cells. For example, nNOS is expressed in neuronal cells (ganglions, trachea and bronchi), and eNOS is expressed in vascular endothelial cells and type ІІ alveolar epithelial cells in humans [4],[5]. On the other hand, the expression of iNOS has not been reported in quiescent cells in healthy subjects, but there have been reported that it is expressed in the airway and the lung parenchyma following stimulation by microbial endotoxins and certain proinflammatory cytokines [4],[5].

Free radicals, including NO, play an important role in the development of pulmonary fibrosis [6]. In fact, increases in the expression of these NOSs in the lungs, and the plasma NOx (nitrite plus nitrate) level, a marker of NO production, have been reported in patients with pulmonary fibrosis [7]–[9]. The roles of the NOS system in the lungs have been evaluated using several types of animal models, and eNOS has been reported to exert a protective role in pulmonary fibrosis [10],[11]. Conflicting results have been reported with regard to iNOS, with some studies showing pathogenic [12]–[14] and protective [15],[16] roles for the enzyme in pulmonary fibrosis. However, because of the different roles of each NOS and the compensatory interactions among these different NOSs [3],[17], the assessment of the roles of NO and the NOSs themselves is difficult, and the roles of the entire NO and NOS system in pulmonary fibrosis remain to be fully elucidated.

Tsutsui *et al*. have developed a mouse model in which all three NOSs were completely deleted [3],[17], and these triple NOS knockout (n/i/eNOS^−/−^) mice demonstrated less than 3% of the normal level of NOx [17]. The authors also reported that n/i/eNOS^−/−^ mice are indistinguishable from wild-type (WT) mice in terms of phenotype and develop normally with a standard increase in body weight. However, they also documented that n/i/eNOS^−/−^ mice are significantly hypertensive compared with WT mice and display characteristics consistent with those of nephrogenic diabetes insipidus [17].

In this study, we investigated the essential roles of NO and the NOS system in a bleomycin (BLM)-induced pulmonary fibrosis model using the n/i/eNOS^−/−^ mice.

## Materials and methods

### Animals

This study was reviewed and approved by the Ethics Committee of Animal Care and Experimentation, University of Occupational and Environmental Health, Japan, and was carried out according to the Institutional Guidelines for Animal Experimentation and the Law (No. 105) and Notification (No. 6) of the Japanese Government. The investigation conforms to the Guide for the Care and Use of Laboratory Animals published by the US National Institutes of Health (NIH Publication No. 85–23, revised 1996). Experiments were performed in seven or eight-week-old male WT (C57/B6) (Kyudo, Co., Ltd., Tosu, Japan), nNOS^−/−^, iNOS^−/−^, eNOS^−/−^ and n/i/eNOS^−/−^ mice weighing 20–25 g [17]. The mice were maintained on a regular diet (CE-2, CLEA Japan, Inc., Tokyo, Japan).

### Animal treatment

Mice were divided into two experimental groups: a BLM-treated group and a control group. BLM (Nippon Kayaku, Tokyo, Japan) was dissolved in 200 μl of normal saline (NS) and administered intraperitoneally at a dose of 8.0 mg/kg/day for 10 consecutive days. For controls, age-matched mice received an identical volume of NS. In the experiment in which the effect of a NO donor on BLM-induced pulmonary fibrosis was examined, the following three groups were studied: WT mice receiving regular drinking water, n/i/eNOS^−/−^ mice receiving drinking water and n/i/eNOS^−/−^ mice receiving isosorbide dinitrate (ISDN, 0.6 mg/dl, Eisai Co., Ltd., Tokyo, Japan) in drinking water from three days before starting BLM administration until sacrifice [18].

### Histopathological evaluation

Two weeks after the last administration of BLM, the body weights of the mice were recorded, and the mice were sacrificed by exsanguination by cutting the axillary artery under deep anesthesia (sodium pentobarbital, 50 mg/kg, i.p.). The left lungs were removed via a midline incision, fixed in 15% formalin neutral buffer solution (Wako, Osaka, Japan) and embedded in paraffin. Then 3-μm sections of embedded tissues were stained with hematoxylin-eosin (HE) and Masson trichrome. The fibrotic area was calculated by microscopy in Masson trichrome-stained sections using an image analysis (BIOREVO BZ-9000 and BZ-H2C; Keyence, Japan), as described previously [19] (see Additional file 1).

### Immunohistochemistry

The immunological detection of macrophages and fibroblasts in the lungs was performed using a rat anti-mouse MAC-2 monoclonal antibody (1:500; Cedarlane Laboratories Ltd, Burlington, ON, Canada) for detecting macrophages, and a monoclonal mouse anti-human smooth muscle actin (α-SMA) antibody (1:150; Dako Cytomation Co, Tokyo) for the detection of fibroblasts [20]. In addition, the immunological detection of connective tissue growth factor (CTGF) and collagen I was performed using rabbit anti-mouse CTGF polyclonal antibodies or collagen I polyclonal antibodies (Abcam, Inc., Cambridge, Mass., USA), according to the manufacturer’s protocol (see Additional file 1).

### Collagen assay

We measured the collagen content in the right lungs of the mice at two weeks after the last administration of BLM using the Sircol Collagen Assay kit (Biocolor Ltd, UK), as reported previously [21] (see Additional file 1).

### Bronchoalveolar lavage

The bronchoalveolar lavage (BAL) was obtained by cannulating the trachea with a 20-gauge catheter. After counting the cell numbers in the BAL fluid (BALF), the cells were cytospun and stained with Diff-Quick for cell classification (see Additional file 1). The cell-free supernatants were stored at −80°C until further analysis. The total protein concentration was also measured using a BIO-RAD Protein Assay Kit ІІ (500-0002JA, Hercules, CA), according to the manufacturer’s protocol.

### Quantitative determination of IL-6, IL-1β, TNF-α, IFN-γ, CCL-2 and active TGF-β1

The concentrations of murine interleukin (IL)-6, IL-1β, tumor necrosis factor (TNF)-α, interferon (IFN)-γ, CC chemokine ligand 2 (CCL-2) and active tissue growth factor-β1 (TGF-β1) in the BALF were determined using ELISA kits (R&D Systems, Minneapolis, MN) according to the manufacturer’s protocol.

### Real-time polymerase chain reaction

Total RNA was extracted from homogenized right lung tissue using the Isogen reagent (Nippon Gene, Tokyo, Japan), and was reverse-transcribed. Quantification of the expression level of each mRNA (IL-6, IL-1β, TNF-α, IFN-γ, CCL-2, TGF-β1, CTGF, collagen I and GAPDH mRNA) was performed by real-time quantitative polymerase chain reaction on an ABI prism 7000 sequence detection system (Applied Biosystems, Foster City, CA) (see Additional file 1).

### NOx measurement

Blood samples were obtained from the right axillary artery at the time of sacrifice, and were immediately centrifuged at 3500 rpm at 4°C for 10 min, and the supernatants were stored at −80°C until they were analyzed. The plasma NOx concentrations were assessed by the Griess method using the ENO-20 NOx analysis system (Eicom, Kyoto, Japan), as reported previously [17],[18].

### Statistical analysis

The statistical analyses were performed using the SPSS software package (version 19), and a value of *P* < 0.05 was considered to be statistically significant. In addition, the Mann–Whitney U (non-parametric) test was used for all statistical analyses.

## Results

### Body weight changes

**Figure 1:**
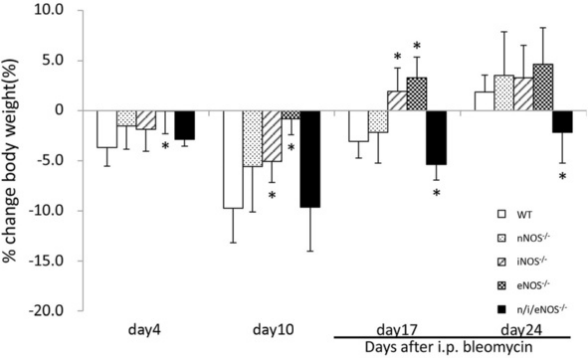
**Temporal changes in the body weight in a pulmonary fibrosis model at two weeks after BLM-treatment (n = 5-7).** The changes in the ratios of the body weights at specific times/initial body weight from the start of BLM administration to the time of sacrifice (day 4, 10, 17 and 24) are shown. *P < 0.05 vs. BLM-treated WT mice.

The average baseline body weights of the mice with various genotypes did not differ significantly (WT, 23.3 ± 0.6 g; nNOS^−/−^, 23.8 ± 0.9 g; iNOS^−/−^, 23.2 ± 0.5 g; eNOS^−/−^, 23.9 ± 1.7 g and n/i/eNOS^−/−^, 22.8 ± 0.6 g). The ratios of body weights at the different times/initial body weight in all of the genotype groups are shown in Figure 1. The WT, single NOS^−/−^ mice, and n/i/eNOS^−/−^ mice exhibited a loss of body weight at the last administration of BLM (day 10). The WT and single NOS^−/−^ mice regained their body weight by two weeks after the last administration of BLM (day 24), whereas significant body weight loss was still observed in the n/i/eNOS^−/−^ mice.

### BLM-induced pulmonary fibrosis

**Figure 2:**
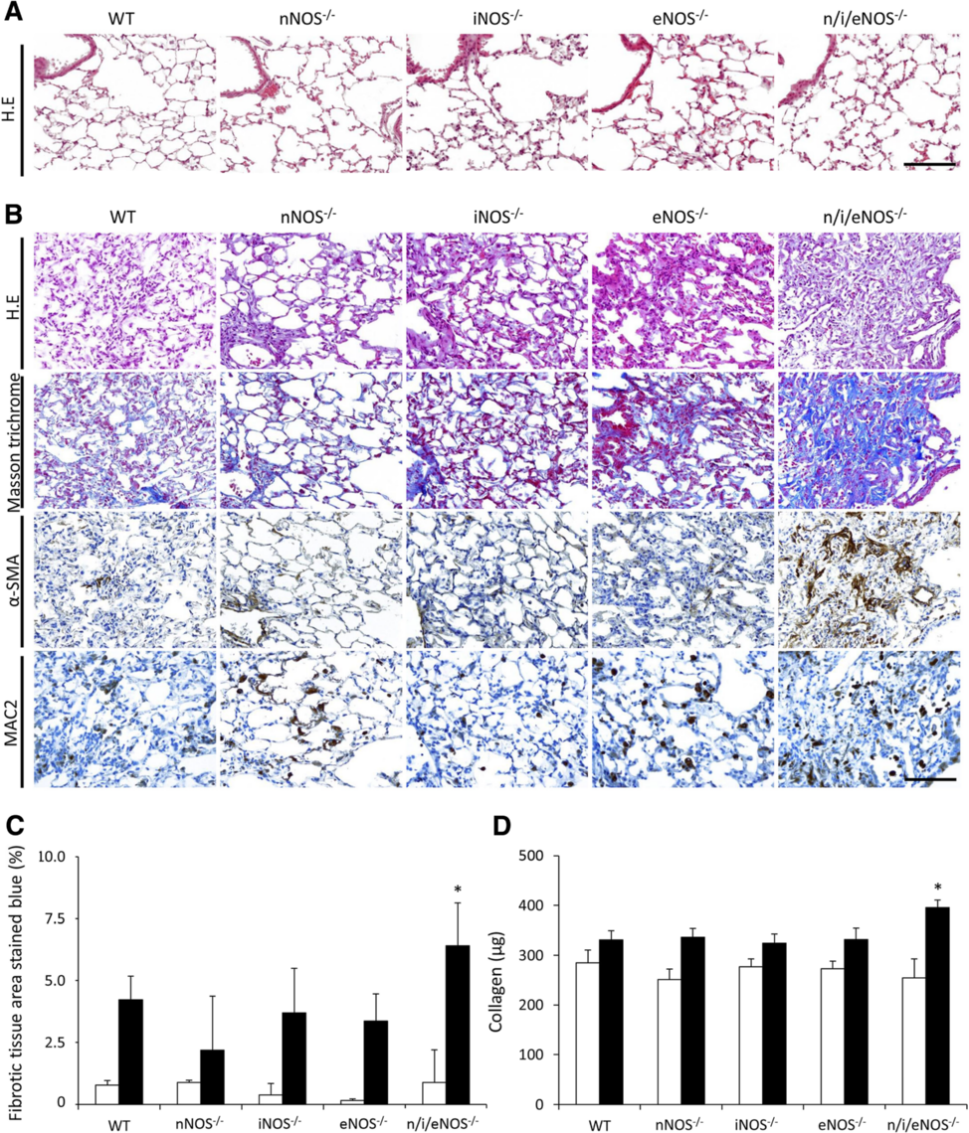
**The n/i/eNOS**^**−/−**^**mice showed a deterioration of lung fibrosis in a pulmonary fibrosis model at two weeks after BLM-treatment. (A)** Hematoxylin-eosin staining in normal saline (NS)-treated mice. Scale bar = 100 μm. **(B)** Hematoxylin-eosin staining, Masson-trichrome staining, α-SMA staining, MAC-2 staining in BLM-treated mice. Scale bar = 100 μm. **(C)** The fibrotic tissue area (blue-stained). **(D)** The collagen content in lung tissue. White and black bars indicate NS- (n= 3) and BLM- (n = 5) treated mice, respectively. *P < 0.05 vs. BLM-treated WT mice.

A histological evaluation revealed no changes in any of the genotype groups in the NS-treated mice (Figure 2A). In contrast, fibrotic changes were obvious in all of the mice at two weeks after the last administration of BLM. The extent of fibrotic changes was the greatest in the n/i/eNOS^−/−^ mice (Figure 2B). On the other hand, there were minimal changes in the WT and single NOS^−/−^ mice, whereas the eNOS^−/−^ mice exhibited more pulmonary cellular infiltration and collagen deposition than the WT mice according to the histological findings (Figure 2B). A quantitative image analysis indicated that a significant increase in the pathological fibrotic tissue area was seen only in the n/i/eNOS^−/−^ mice, and no significant differences were observed among the WT and single NOS^−/−^ mice (Figure 2C). Furthermore, the collagen assay demonstrated that the amount of collagen was the greatest in the n/i/eNOS^−/−^ mice, while there were no significant differences among the WT and single NOS^−/−^ mice (Figure 2D).

### Total cell counts and differential cell analysis of the BALF

**Figure 3:**
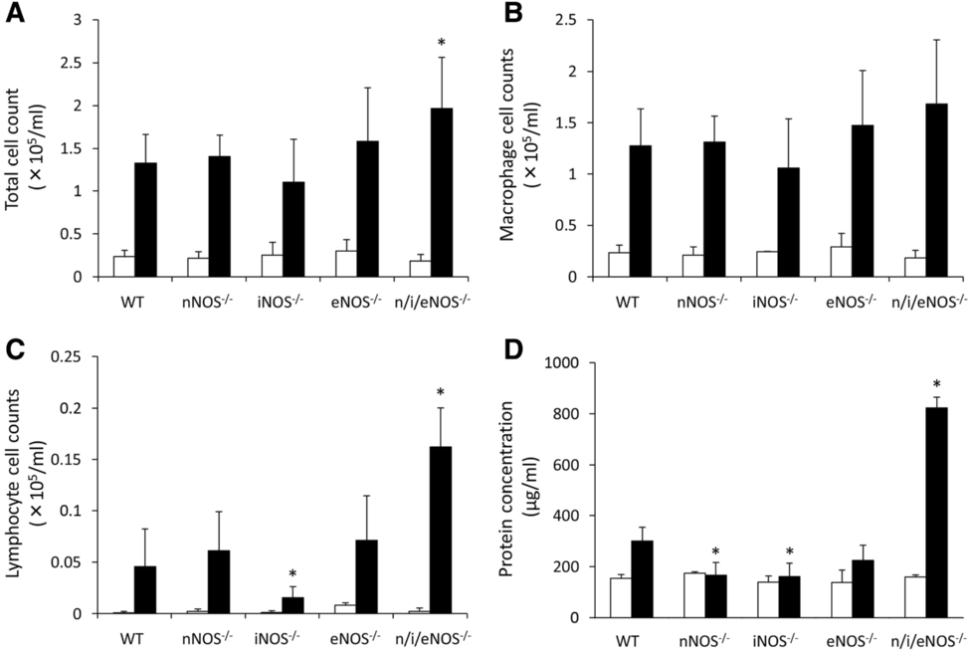
**The n/i/eNOS**^**−/−**^**mice showed an increase in the number of inflammatory cell in the bronchoalveolar lavage fluid in a pulmonary fibrosis model at two weeks after BLM-treatment. (A)** The total cell counts. **(B)** The macrophage cell counts. **(C)** The lymphocyte cell counts. **(D)** The total protein concentrations. White and black bars indicate normal saline- (n = 3) and BLM-(n = 5) treated mice, respectively. *P < 0.05 vs. BLM-treated WT mice.

The total cell counts and differential cell counts in the BALF were analyzed at two weeks after the last administration of BLM. The mean total cell counts obtained from n/i/eNOS^−/−^ mice were significantly higher than those of all of the other genotypes (Figure 3A), and the cell counts of lymphocytes obtained from n/i/eNOS^−/−^ mice were also significantly higher than those of WT and single NOS^−/−^ mice (Figure 3C). On the other hand, there were no significant differences between the cell counts of macrophages in any of the genotype groups (Figure 3B). The total protein concentration in the n/i/eNOS^−/−^ mice was also significantly higher than that of the WT and single NOS^−/−^ mice (Figure 3D).

In addition, there were no significant changes in the total cell counts and macrophage counts between WT and single NOS^−/−^ mice (Figure 3A and B), but the cell counts of lymphocytes in the iNOS^−/−^ mice was significantly lower than that of the WT mice (Figure 3C). The total protein concentration in the nNOS^−/−^ and iNOS^−/−^ mice was also significantly lower than that of the WT mice (Figure 3D).

### Quantitative analysis of the protein levels and the mRNA expression of pro-inflammatory cytokines

**Figure 4:**
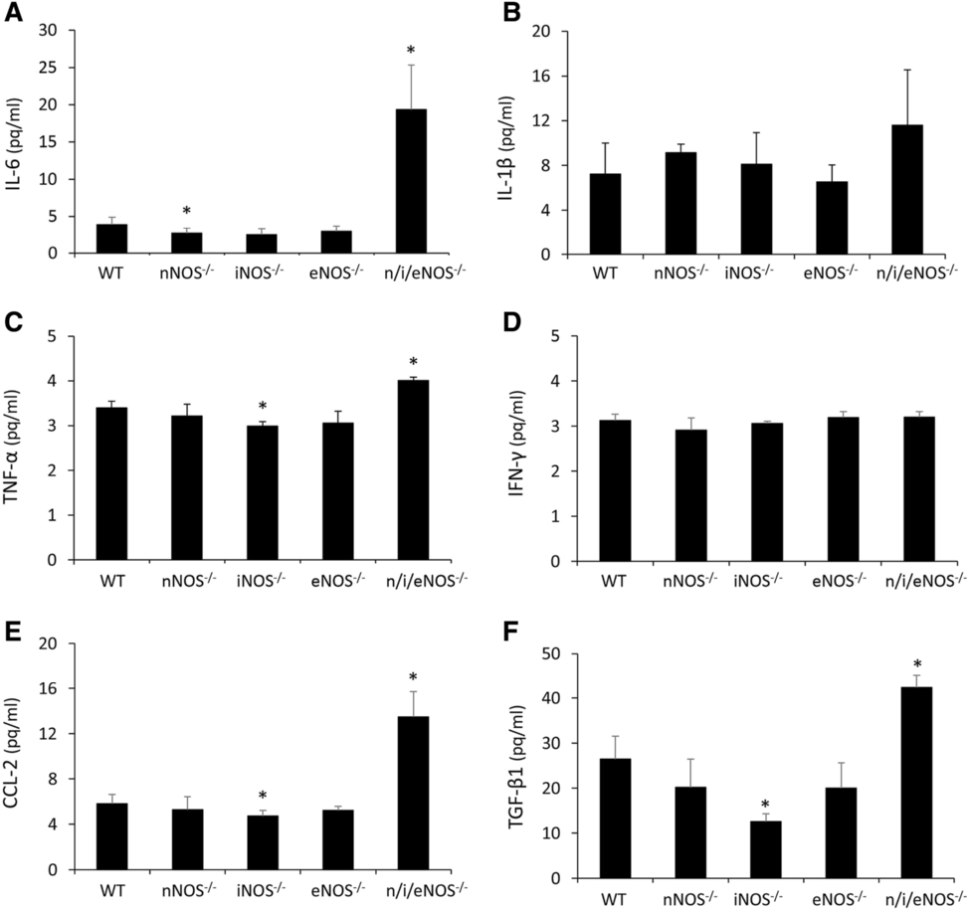
**The n/i/eNOS**^**−/−**^**mice were associated with an increase in the protein levels of proinflammatory cytokines, CC chemokine ligand 2 (CCL-2), and the tissue growth factor-β1 (TGF-β1) in a pulmonary fibrosis model at two weeks after BLM-treatment in BALF (n = 5). (A)** IL-6 protein. **(B)** IL-1β protein. **(C)** TNF-α protein. **(D)** IFN-γ protein. **(E)** CCL-2 protein. **(F)** Active form of TGF-β1. *P < 0.05 vs. BLM-treated WT mice.

**Figure 5:**
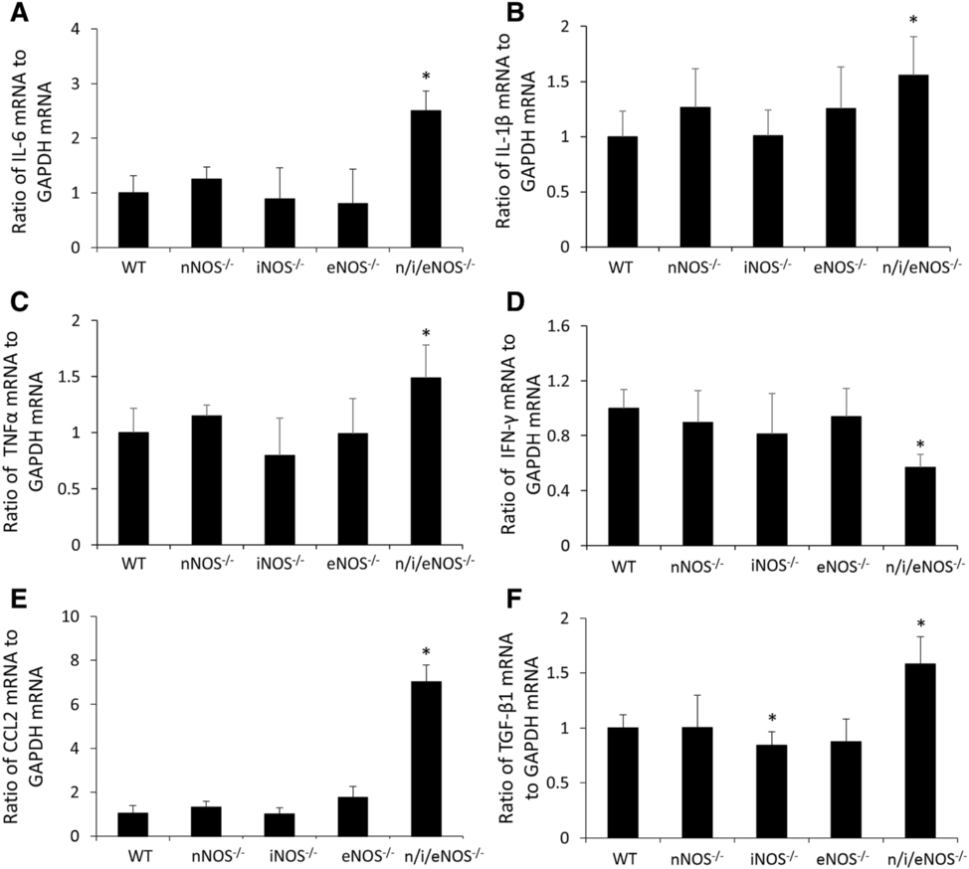
**The mRNA expression of pro-inflammatory cytokines, CC chemokine ligand 2 (CCL-2), and the tissue growth factor-β1 (TGF-β1) in the lung in a pulmonary fibrosis model at two weeks after BLM-treatment (n = 5). (A)** IL-6 mRNA expression. **(B)** IL-1β mRNA expression. **(C)** TNF-α mRNA expression. **(D)** IFN-γ mRNA expression. **(E)** CCL-2 mRNA expression. **(F)** TGF-β1 mRNA expression. *P < 0.05 vs. BLM-treated WT mice.

The protein levels of IL-6 and TNF-α were significantly higher in the n/i/eNOS^−/−^ mice than in the WT mice at two weeks after the last administration of BLM (Figure 4A and C). The expression of IL-6, IL-1β and TNF-α mRNA in the n/i/eNOS^−/−^ mice was also significantly higher than that in the WT mice (Figure 5A-C) at two weeks after the last administration of BLM. On the other hand, the expression of IFN-γ mRNA in the n/i/eNOS^−/−^ mice was significantly lower than that of the WT mice, although the protein level of IFN-γ demonstrated no significant change (Figures 4D and 5D). There were no obvious differences in the protein (Figure 4A-D) or mRNA (Figure 5A-D) levels between the WT and single NOS^−/−^ mice.

### Quantitative analysis of the protein level and the mRNA expression of CCL-2

The protein level of CCL-2 (Figure 4E) and the expression of CCL-2 mRNA (Figure 5E) were significantly higher in the n/i/eNOS^−/−^ mice compared to the WT mice at two weeks after the last administration of BLM. The protein level of the iNOS^−/−^ mice was significantly lower than that of the WT mice. (Figure 4E), but there weren’t significant differences in the mRNA levels between the WT and single NOS^−/−^ mice (Figure 5E).

### Quantitative analysis of the active form of TGF-β1 protein and the expression of TGF-β1 mRNA

The protein level of the active form of TGF-β1 of the BALF (Figure 4F) and the expression of TGF-β1 mRNA of the lung (Figure 5F) were significantly higher in the n/i/eNOS^−/−^ mice than in the WT mice at two weeks after the last administration of BLM. Comparing the WT and single NOS^−/−^ mice, the protein and mRNA levels were significantly lower in the iNOS^−/−^ mice than in the WT mice (Figures 4F and 5F).

### Immunochemical expression and the mRNA expression of growth factor

**Figure 6:**
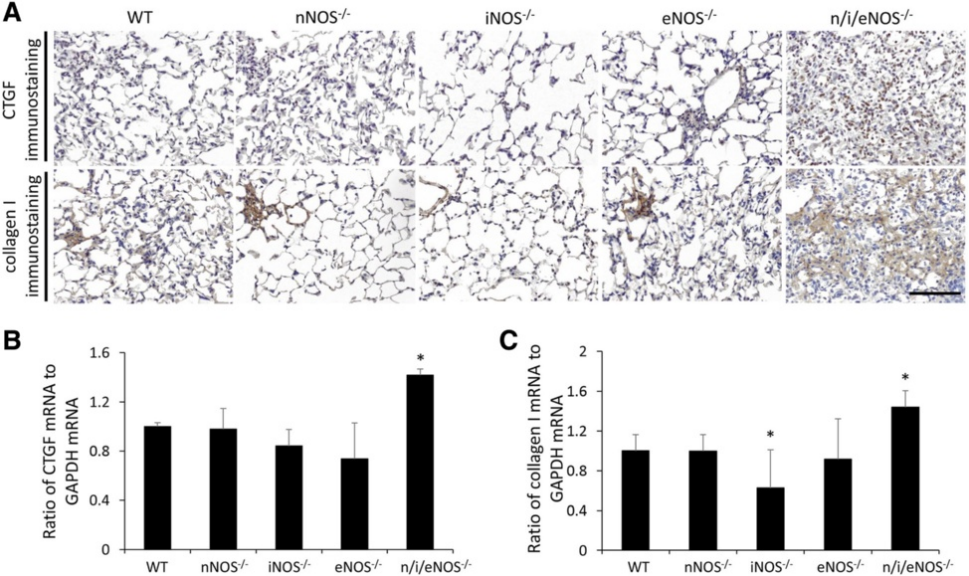
**The n/i/eNOS**^**−/−**^**mice exhibited an increased production of CTGF and collagen I. (A)** Immunostaining for CTGF and collagen I in the lungs of the WT and n/i/eNOS^−/−^ mice. Scale bar = 100 μm. **(B)** CTGF mRNA expression (n = 5). **(C)** Collagen I mRNA expression (n = 5).

Figure 6A shows representative immunohistochemical findings for growth factor. The expression of CTGF and collagen I was higher in the n/i/eNOS^−/−^ mice than in the WT or single NOS^−/−^ mice. And the expression of CTGF and collagen I mRNA in the n/i/eNOS^−/−^ mice were significantly higher than those in the WT mice (Figure 6B and C) at two weeks after the last administration of BLM. Comparing the WT and single NOS^−/−^ mice, mRNA levels of collagen I in the iNOS^−/−^ mice were significantly lower than in the WT mice (Figure 6B and C).

### Effects of long-term supplementation of a NO donor

**Figure 7:**
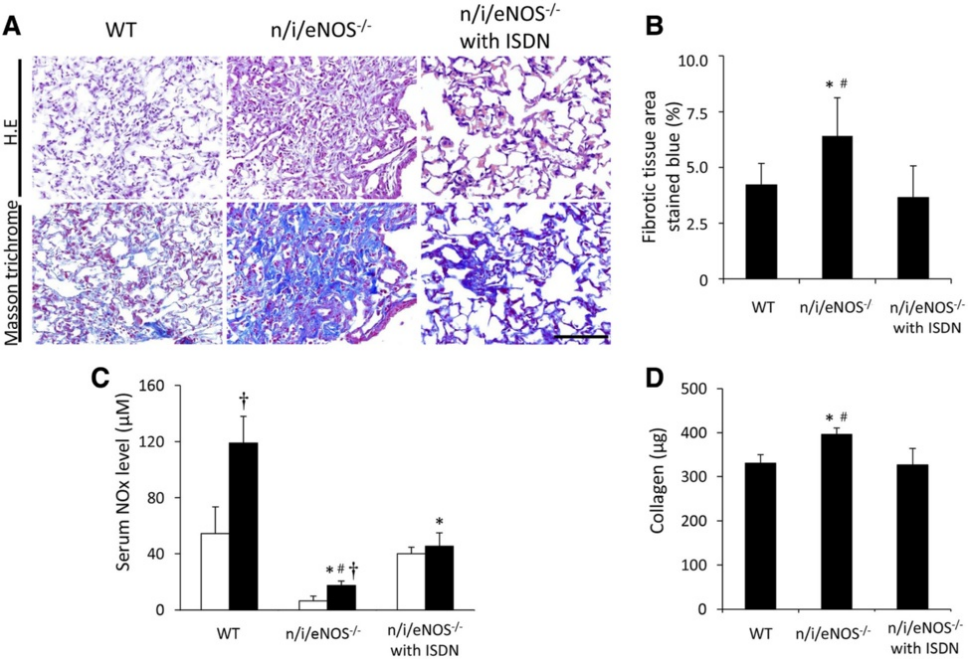
**The anti-fibrotic effects of long-term treatment with a NO donor in a pulmonary fibrosis model at two weeks after BLM-treatment (n = 4-5). (A)** Hematoxylin-eosin staining, Masson-trichrome staining. Scale bar = 100 μm. **(B)** The fibrotic tissue area (blue-stained). **(C)** The serum NOx levels. White and black bars indicate normal saline (NS)- and BLM- treated mice, respectively. **(D)** The collagen content in lung tissue. *P < 0.05 vs. the BLM-treated WT mice. ^#^*P* < 0.05 vs. the BLM-treated n/i/e NOS^−/−^ mice that received ISDN. ^†^*P* < 0.05 vs. the NS-treated mice.

The serum NOx levels were markedly reduced in both NS- and BLM-treated n/i/eNOS^−/−^ mice compared with those in the NS-treated WT mice (Figure 7C). Long-term oral administration of ISDN significantly restored the NOx levels in both NS- and BLM-treated n/i/eNOS^−/−^ mice up to the levels observed in NS-treated WT mice (Figure 7C). Additionally, the long-term treatment with ISDN significantly prevented the progression of the histological findings and the increase in collagen content in the n/i/eNOS^−/−^ mice (Figure 7A, B, and D).

## Discussion

In the present study, we evaluated the roles of NO and the NOS system in pulmonary fibrosis by using mice lacking all three NO synthases, n/i/eNOS^−/−^ mice, and showed that the lack of all NOS led to a deterioration of the fibrotic changes in the lungs of mice with BLM-induced pulmonary fibrosis. In addition, these findings were prevented by long-term treatment with a NO donor, ISDN. This is the first report to show that NO is an important factor in the progression of pulmonary fibrosis, and that NO has protective effects against BLM-induced pulmonary fibrosis.

With regard to the roles of NO in the progression of fibrosis, there have been several reports showing the protective roles of NO in cardiac [22] and renal [23] fibrosis using non-selective NOS inhibitors in mouse models. So far, a non-selective NOS inhibitor has been reported to worsen the mortality in a BLM-induced murine pulmonary fibrosis model [10] and accelerated pulmonary granuloma formation in a purified protein derivative murine model [24], another model of pulmonary fibrosis. However, because of the non-specificity of these inhibitors [25],[26], it is difficult to evaluate the essential roles of NO. Therefore, little has been known about the functions and roles of NO itself in pulmonary fibrosis. Concerning the role of each NOS isoform in pulmonary fibrosis, the protective effects of pulmonary fibrosis in eNOS transgenic mice [10] and the deterioration of fibrosis in eNOS^−/−^ mice [11] have also been reported. The inhibition of iNOS has been reported to suppress pulmonary fibrosis in murine model using iNOS^−/−^ mice [12] and mice treated with a selective iNOS inhibitor [12]–[14], but there have been several conflicting reports that iNOS^−/−^ led to a deterioration of the progression of pulmonary fibrosis [15],[16]. It has been reported that the expression of nNOS was unchanged in a BLM-inhalation rat model [27], and the role of nNOS in pulmonary fibrosis has not been fully understood.

Therefore, the role of NO in pulmonary fibrosis has been controversial, mainly because each isoform has different functions and compensatory interactions with the other isoforms [3],[17]. The n/i/eNOS^−/−^ mice provide one way to resolve the former problems of the animal models using single NOS^−/−^ mice or various NOS inhibitors, and we believe this murine model is an important tool for understanding the essential roles of NO [18],[28].

NO and the NOS system have been suggested to have both beneficial and deleterious effects on the respiratory system [4]. These results are confusing with respect to understanding the essential role of NO. In the present study, the BLM-treated WT mice demonstrated increased NOx concentrations as well as a deterioration of fibrotic changes compared with that observed in the NS-treated WT mice, as well as increased plasma NOx levels have been reported in patients with pulmonary fibrosis [9]. While the BLM-treated n/i/eNOS^−/−^ mice, despite the lack of NOx, exhibited a significant deterioration of fibrotic changes compared to the BLM-treated WT mice. In addition, the poor factors observed in the BLM-treated n/i/eNOS^−/−^ mice were prevented via ISDN treatment by increasing the NOx levels up to that observed in the NS-treated WT mice. Therefore, we believe that strongly reduced concentrations of NO may be associated with the progression of BLM-induced pulmonary fibrosis and that an appropriate NO concentration is required for respiratory homeostasis.

In addition, a significant body weight loss has been reported in parallel with a deterioration of pulmonary fibrosis in a BLM-treated mouse model [29]. In the present study, the BLM-treated n/i/eNOS^−/−^ mice also exhibited a significant protracted course of body weight loss compared with the WT and single NOS^−/−^ mice.

TGF-β1 is an important pathogenic factor involved in a variety of fibroproliferative disorders, including pulmonary fibrosis [1],[13], and there have been several *in vitro* reports that showed an increased expression of NO and a subsequent decrease of TGF-β1 due to the increase of NO production [13],[30]. Shibata *et al.* reported an elevation of cardiac TGF-β1 expression in n/i/eNOS^−/−^ mice [31], and we similarly observed upregulation of the protein levels and mRNA of pulmonary TGF-β1 in BLM-treated n/i/eNOS^−/−^ mice in this study. It is well known that TGF-β1 promotes the production of CTGF [32] and collagen I [33], which leads to the progression of pulmonary fibrosis. The subsequent increased production of CTGF and collagen I was noted in the BLM-treated n/i/eNOS^−/−^ mice in this study. From these results, with respect to the mechanisms underlying the antifibrotic activity induced by the absence of NO, the TGF-β1/CTGF pathway is one possible pathway involved in this process. CTGF is considered to play a critical role in the onset of fibrosis as a downstream mediator of TGF-β1 [34], and the downregulation of the expression of CTGF mRNA by NO donors in rat mesangial cells has been previously reported [34]. NO has also been reported to suppress the expression of CTGF by inhibiting Smad-dependent TGF-β signaling [35]. Taken together, the deterioration of pulmonary fibrosis in the BLM-treated n/i/eNOS^−/−^ mice observed in this study may be explained by the above mechanism, although further studies are needed to clarify the mechanisms underlying the antifibrotic activity of NO in the setting of fibrotic lung diseases.

It is well known that increased expression levels of the proinflammatory cytokines IL-6, IL-1β and TNF-α and decreased expression levels of the anti-fibrotic cytokine IFN-γ are involved in the pathogenesis and progression of pulmonary fibrosis [36],[37]. Our present results are consistent with the findings of former reports, although there were no significant differences in the protein levels of IL-1β or IFN-γ. Considering the relationships between NO and the above proinflammatory cytokines, NO has been reported to be a potent inhibitor of the proinflammatory cytokine production induced by alveolar macrophages [38],[39]. Therefore, the increased levels of pulmonary inflammatory cytokines (IL-6, IL-1β and TNF-α) observed in the BLM-treated n/i/eNOS^−/−^ mice in the present study may also be explained by an increase in proinflammatory cytokine production stimulated by alveolar macrophages. Therefore, we speculate that alveolar macrophages are potent targets in the deterioration of pulmonary fibrotic changes associated with the absence of NO.

CCL-2 was upregulated in BLM-treated n/i/eNOS^−/−^ mice compared to BLM-treated WT mice, and therefore, the CCL-2/NO pathway was considered as an alternative pathway leading to BLM-induced pulmonary fibrosis in this study. CCL-2, also known as monocyte chemotactic protein-1 (MCP-1), belongs to the C-C chemokine superfamily of small proteins, and is considered to be a potent chemoattractant for monocytes/macrophages. Several reports have demonstrated that CCL-2 plays an important role in the development of pulmonary inflammation and fibrosis in both animal models [40] and human studies [41]. Previous *in vitro* and *in vivo* studies have shown that endothelial NO synthesis was inhibited by a non-selective NOS inhibitor and this inhibition of endothelial NO synthesis led to an increase in CCL-2 expression [42],[43]. The production of TGF-β1 induced by CCL-2 has also been reported *in vitro*[44], and the promotion of TGF-β1 production may be explained by the increased CCL-2 production in BLM-treated n/i/eNOS^−/−^ mice in this study.

In this study, the eNOS^−/−^ mice treated with BLM histopathologically exhibited more cellular infiltration and collagen deposition than the WT mice, although the findings of the quantitative evaluation of the fibrotic areas and collagen deposition and the analyses of the BALF did not differ significantly from those observed in the WT mice. The protective effects of eNOS against pulmonary fibrosis have been demonstrated in various studies [10],[11], and we believe that eNOS may also protect against the development of pulmonary fibrosis. However, it was not possible to elucidate the role of each NOS isoform in fibrotic changes compared to the WT mice based on the results of this study. These results were similar to the previous reports in models of carotid artery ligation or a high-cholesterol diet [18],[28]. Compensatory mechanisms involving other NOSs in terms of producing NO may explain these findings. Indeed, Morishita *et al.* have revealed that the other NOSs are highly expressed in the single NOS^−/−^ and double NOS^−/−^ mice, and that NOx production is fairly well preserved in mice of those genotypes [17]. These findings may support the importance of using a murine model lacking all three types of NOS when investigating the true functions of NO.

In conclusion, we provide the first evidence that a lack of all three NO synthases leads to the deterioration of fibrotic changes in BLM-induced pulmonary fibrosis in mice. It is speculated that NO plays an important protective role in the pathogenesis of pulmonary fibrosis.

## Abbreviations

NO: Nitric oxide

NOS: Nitric oxide synthase

BLM: Bleomycin

ISDN: Isosorbide dinitrate

HE: Hematoxylin-eosin

BAL: Bronchoalveolar lavage

BALF: Bronchoalveolar lavage fluid

TGF-β1: Tissue growth factor-β1

CCL-2: CC chemokine ligand 2

IL: Interleukin

TNF-α: Tumor necrosis factor-α

IFN-γ: Interferon-γ

CTGF: Connective tissue growth factor

## Competing interests

All the authors report no potential conflicts of interest.

## Authors’ contributions

SN (designed experiments, performed data analysis, wrote the first draft), KY (designed experiments, performed data analysis, provided intellectual contributions), WKY (designed experiments, provided intellectual contributions), KO (provided intellectual contributions), KA (provided intellectual contributions), KY (designed experiments, provided intellectual contributions), TK (provided intellectual contributions), HI (provided intellectual contributions), YT (provided intellectual contributions), HS (provided intellectual contributions), NY (provided intellectual contributions), MT (provided intellectual contributions), HM (conceived & designed experiments, provided intellectual contributions). All authors read and approved the final manuscript.

## Additional file

## Supplementary Material

Additional file 1: **Detailed description of**Materials and methods section. **Figure S1.** Immunostaining for nNOS, iNOS, eNOS in the lung of the WT and n/i/eNOS^−/−^ mice treated with normal saline or BLM. **Table S1.** Primers and probes used for real-time PCR. Click here for file
