# Supplementary material for: Nitric oxide exerts protective effects against bleomycin-induced pulmonary fibrosis in mice
Source: Respir Res. 2014 Aug 5;15(1):92. doi: 10.1186/s12931-014-0092-3 (PMC4237963; doi:10.1186/s12931-014-0092-3)
Supplement: Additional file 1: — Detailed description ofMaterials and methods section. Figure S1. Immunostaining for nNOS, iNOS, eNOS in the lung of the WT and n/i/eNOS−/− mice treated with normal saline or BLM. Table S1. Primers and probes used for real-time PCR. [file s12931-014-0092-3-S1.docx]

Additional file 1 – Detailed description of Materials and Methods section

**Nitric oxide exerts protective effects against bleomycin-induced pulmonary fibrosis in mice**

Shingo Noguchi, Kazuhiro Yatera, Ke-Yong Wang, Keishi Oda, Kentarou Akata, Kei Yamasaki, Toshinori Kawanami, Hiroshi Ishimoto, Yumiko Toyohira, Hiroaki Shimokawa, Nobuyuki Yanagihara, Masato Tsutsui, Hiroshi Mukae

**Materials and Methods**

**(full versions for the online supplemental materials)**

**Histopathological evaluation**

Two weeks after the last administration of BLM, the body weights were recorded and the mice were sacrificed by exsanguination by cutting the axillary artery under deep anesthesia (sodium pentobarbital, 50 mg/kg, i.p.). The left lungs were removed via a midline incision, fixed in 15% formalin neutral buffer solution (Wako, Osaka, Japan) and embedded in paraffin, then 3-μm sections of embedded tissues were stained with hematoxylin-eosin (HE) and Masson trichrome stain. The fibrotic areas were calculated by performing a microscopic examination of the Masson trichrome-stained sections using an image analysis software program (BIOREVO BZ-9000 and BZ-H2C; Keyence, Japan) [1]. This software program automatically measures the area of a designated color in a section. The criteria for the blue Masson trichrome stain was appropriately determined and saved by the computer. The saved quantification condition was applied for all images. Then, the ratio of the area stained blue to the lung area in each section was evaluated.

**Immunohistochemistry**

The immunological detection of nNOS, iNOS, and eNOS was performed using a rabbit polyclonal antibody (Transduction Laboratories, Franklin Lakes, NJ) at a dilution of 1:500 for nNOS, 1:100 for iNOS, and 1:250 for eNOS [2]. Next, the immunological detection of macrophages and fibroblasts in the lungs was performed using a rat anti-mouse MAC-2 monoclonal antibody (1:500; Cedarlane Laboratories Ltd, Burlington, ON, Canada) for detecting macrophages, and a monoclonal mouse anti-human smooth muscle actin (α-SMA) antibody (1:150; Dako Cytomation Co, Tokyo) for the detection of fibroblasts [3]. In addition, the immunological detection of connective tissue growth factor (CTGF) and collagen I was performed using CTGF or collagen I antibody ( Abcam, Inc., Cambridge, Mass., USA), according to the manufacturer’s protocol.

**Collagen assay**

We measured the collagen content in the right lungs of the mice two weeks after the last administration of BLM using the Sircol Collagen Assay kit (Biocolor Ltd, UK). [4]. In brief, each lung was homogenized in 10 ml of 0.5 M acetic acid containing pepsin (0.1 mg/ml). Each homogenized sample was incubated for 24 h at 4°C with stirring. After centrifugation, 1 ml of each supernatant was assayed. One milliliter of Sircol dye reagent, which binds to collagen, was added to each sample and then mixed for 30 min. After centrifugation, the pellet was suspended in 1 ml of the alkali reagent included in the kit, and the absorbance of the sample was read at 555 nm by a spectrophotometer. Collagen standard solutions were utilized to construct a standard curve.

**Bronchoalveolar lavage**

Bronhoalveolar lavage fluid (BALF) was obtained by cannulating the trachea with a 20- gauge catheter. An aliquot of saline (0.9% NaCl) was injected into the lung, and a total volume of 5 ml was recovered. The cell-free supernatants were stored at −80°C until further analysis. The recovered fluid was filtered, and 100 µml of this filtered sample was mixed with 100 µml of Türk's Solution, then the cells were counted under a microscope (OLYMPUS BX51, Tokyo, Japan). Differential cell counts were determined using the cell suspensions displayed on glass slides using a cytocentrifuge (Cytospin 4; Termo, Kanagawa, Japan). The cells on a slide were dried, fixed and then stained by the Diff-Quick (Sysmex, Hyogo, Japan) method, then one hundred cells were identified under a microscope. The total protein concentration was also measured using a BIO-RAD Protein Assay Kit ІІ (500-0002JA, Hercules, CA).

**Real-time polymerase chain reaction**

For RNA isolation, the right lungs were stored at −20°C immediately after removal from each mouse. The total RNA was extracted from homogenized right lung tissues using the Isogen reagent (Nippon Gene, Tokyo, Japan), and was reverse-transcribed according to the instructions provided by the manufacturer. A volume of pooled RNA (1 μl) was reverse-transcribed in a total reaction volume of 21 μl containing 0.5 μg of random hexamer primers, 20 units RNasin ribonuclease inhibitor and SuperScript III First-Strand Synthesis System (Invitrogen), according to the supplier’s instructions. The resulting cDNA was subjected to quantitative real-time polymerase chain reaction (QRT-PCR). The QRT-PCR amplification was performed by real-time quantitative polymerase chain reaction (PCR) on an ABI prism 7000 sequence detection system (Applied Biosystems, Foster City, CA). The PCR conditions were: 95°C for 15 min, followed by 40 cycles of 15 s at 94°C and 60 s at 60°C. The value of each hybridized probe was normalized to that of GAPDH in each template, which was set as an internal control. The primers for TGF-β1, CCL-2, connective tissue growth factor (CTGF), collagen I and GAPDH mRNA were included in the Taq-Man Gene Expression Assays (Applied Biosystems, Foster City, CA). The sequences of the specific primers used for interleukin (IL)-6, IL-1β, tumor necrosis factor (TNF)-α and interferon (IFN)-γ mRNA are summarized in Table S1.

**References**

1. Mizuta M, Hirano S, Hiwatashi N, Tateya I, Kanemaru S, Nakamura T, Ito J: **Effect of astaxanthin on vocal fold wound healing.** *Laryngoscope* 2014, **124:**E1-7.

2. Furuno Y, Morishita T, Toyohira Y, Yamada S, Ueno S, Morisada N, Sugita K, Noguchi K, Sakanashi M, Miyata H, Tanimoto A, Sasaguri Y, Shimokawa H, Otsuji Y, Yanagihara N, Tamura M, Tsutsui M: **Crucial vasculoprotective role of the whole nitric oxide synthase system in vascular lesion formation in mice: Involvement of bone marrow-derived cells.** *Nitric Oxide* 2011, **25:**350-359.

3. Tasaki T, Yamada S, Guo X, Tanimoto A, Wang KY, Nabeshima A, Kitada S, Noguchi H, Kimura S, Shimajiri S, Kohno K, Ichijo H, Sasaguri Y: **Apoptosis signal-regulating kinase 1 deficiency attenuates vascular injury-induced neointimal hyperplasia by suppressing apoptosis in smooth muscle cells.** *Am J Pathol* 2013, **182:**597-609.

4. Tokuda A, Itakura M, Onai N, Kimura H, Kuriyama T, Matsushima K: **Pivotal role of CCR1-positive leukocytes in bleomycin-induced lung fibrosis in mice.** *J Immunol* 2000, **164:**2745-2751.

**Figure S1 Immunostaining for nNOS, iNOS, eNOS in the lung of the WT and n/i/eNOS^−/−^ mice treated with normal saline or BLM.**

Among the WT mice, immunoreactivity for nNOS was almost completely absent in the normal saline (NS)-treated mice, while it was faintly observed in the bleomycin (BLM)-treated mice in the macrophages and alveolar epithelium. Immunoreactivity for iNOS was observed in the BLM-treated WT mice in the macrophages and alveolar epithelium, but it was also almost absent in the NS-treated mice. The eNOS immunoreactivity was also faintly noted in the NS- and BLM-treated mice in the macrophages and alveolar epithelium. In contrast, no immunoreactivity was noted in the lungs of the NS- or BLM-treated n/i/eNOS^-/-^ mice. Scale bar=100µm.

**Table S1. Primers and probes used for real-time PCR**

| Oligo | Sequence | |
| --- | --- | --- |
| IL-6 | |  |
|  | Forward | 5´-TTACACATGTTCTCTGGGAAATCG-3´ |
|  | Reverse | 5´-TTGGTAGCATCCATCATTTCTTTG-3´ |
|  | Probe | 5´-TGAGAAAAGAGTTGTGCAATGGCAATTCTGAT-3´ |
| IL-1β | |  |
|  | Forward | 5´-TGCACTACAGGCTCCGAGATG-3´ |
|  | Reverse | 5´-GTACAAAGCTCATGGAGAATATCACTTG-3´ |
|  | Probe | 5´-TGTCGGACCCATATGAGCTGAAAGCTCTC-3´ |
| TNF-α | |  |
|  | Forward | 5´-CCCAGACCCTCACACTCAGATC-3´ |
|  | Reverse | 5´-TGCTCCTCCACTTGGTGGTT-3´ |
|  | Probe | 5´-ATTCGAGTGACAAGCCTGTAGCCCACG-3´ |
| IFN-γ | |  |
|  | Forward | 5´-CATTCATGAGTATTGCCAAGTTTGAC-3´ |
|  | Reverse | 5´-GCTTCCTGAGGCTGGATTCC-3´ |
|  | Probe | 5´-CCACAGGTCCAGCGCCAAGCATT-3´ |
